# Effectiveness of Combination of Tibolone and *Lactobacilli* Plus Lactoferrin in Postmenopausal Women with Vulvar Vestibular Pain: A Preliminary Report

**DOI:** 10.3390/nu16142378

**Published:** 2024-07-22

**Authors:** Vincenzo De Leo, Laura Governini, Rosetta Ponchia, Dario Recalcati, Filippo Murina

**Affiliations:** 1 Medical Office “Colledoro”, 53100 Siena, Italy; 2 Department of Molecular and Developmental Medicine, University of Siena, 53100 Siena, Italy; ponchia2@student.unisi.it; 3 Lower Genital Tract Disease Unit, V. Buzzi Hospital, University of the Study of Milan, 20124 Milan, Italyfilippo.murina@unimi.it (F.M.)

**Keywords:** vestibulodynia, vulvar pain, tibolone, *Lactobacilli*, lactoferrin

## Abstract

Background: Postmenopausal dyspareunia and vulvar pain are common complaints, affecting about 60% of women within a few years after hormone levels begin to decline (such as estrogen and androgen). Atrophic changes mainly located in the vulvar vestibule and vulnerability to vulvovaginal infections in postmenopause could be predisposing factors to the development of vulvar burning/pain and introital dyspareunia (vestibulodynia secondary to atrophy). Tibolone is the most effective and safe alternative for treating menopausal symptoms. The role of *Lactobacilli* and lactoferrin shows its effectiveness in the treatment of vaginal microbiota dysbiosis. The aim of the present study was to assess the efficacy of the combination of tibolone and an oral-specific *Lactobacilli* mixture in combination with bovine lactoferrin as synergistic therapy for the treatment of vestibulodynia related to atrophy. Methods: In this study, we included 35 postmenopausal women with at least 1 year of amenorrhea, affected by vulvar burning/pain and introital dyspareunia. All participants received treatment with open-label, oral Tibolone 2.5 mg and *Lactobacilli* mixture (5 × 10^9^ CFU per capsule) in combination with bovine lactoferrin (Respecta^®^). Each product was taken once daily for 90 days. Results: After 90 d of therapy with TIB+ Respecta^®^, in 30 women that completed the treatment, there was a statistically significant decrease from the baseline in the mean of the Visual Analog Scale for vulvar burning/pain and a reduction in scores in the pain evaluation test. Conclusions: This study provides evidence that the combination of TIB+ Respecta^®^ was effective in reducing symptoms related to vestibular pain and hypersensitivity in a postmenopausal setting.

## 1. Introduction

Female sexual dysfunction is a prevalent factor contributing to family breakdowns. Recently, multiple strategies have been proposed to address this issue. Dyspareunia and vulvodynia are genital pain disorders that severely impact women’s quality of life. These conditions are highly prevalent and impose a significant financial burden on both women and the healthcare system. Many women do not report experiencing genital pain, and most healthcare providers do not routinely inquire about it. Consequently, women often suffer from social isolation. Although various treatments are believed to improve quality of life and reduce pain, further research is necessary to substantiate their efficacy [1,2,3].

Postmenopausal dyspareunia and vulvar pain are common complaints, affecting about 60% of women within a few years after hormone levels begin to decline (such as estrogen and androgen), which occurs due to menopause [4,5,6]. Vulvodynia is a pathological condition that predominantly affects the vulva and the tissues surrounding the vaginal introitus. Beyond causing pain and other physical symptoms, it significantly impairs women’s quality of life, particularly impacting marital and sexual relationships. Over time, it can also lead to discomfort, anxiety, and frustration, negatively influencing self-esteem and resulting in reduced sexual desire and emotional distancing from partners. Unfortunately, vulvodynia is often underestimated, partly due to women’s reluctance to discuss symptoms out of shame or fear of judgment, and partly due to the diagnostic challenges faced by some healthcare providers. Historically, it was frequently diagnosed late and went untreated for years. It was also considered an “invisible” disease, often misclassified as “psychosomatic” or even “psychogenic”. However, this is a partial and sometimes incorrect interpretation, now considered obsolete. Vulvodynia is a condition with well-established biological foundations that falls within the domain of gynecology and other medical disciplines and can be effectively managed with an appropriate therapeutic protocol.

Vulvovaginal atrophy pertains to the physical changes observed in the vulva and vagina after menopause, yet it fails to encompass the associated symptoms. The term Genitourinary Syndrome of Menopause (GSM) is recommended for a more comprehensive description of the condition [7,8,9]. GSM can encompass genital symptoms such as dryness and burning and sexual symptoms like insufficient lubrication, discomfort or pain, and impaired function. Additionally, urinary symptoms such as urgency, dysuria, and other recurrent disorders may be present. Women may exhibit some or all of these signs and symptoms. The main objectives in managing GSM are to relieve symptoms and reverse atrophic changes. Estrogen therapy, delivered either vaginally or systemically, is regarded as the standard treatment for moderate to severe vulvovaginal symptoms [7,8,10]. However, for many patients, this treatment may not sufficiently reduce dyspareunia or sensitivity, discomfort, or pain associated with vestibular touch or pressure. This limitation might be due to the unique characteristics of the vulvar vestibule.

The vulvar vestibule, a thin band of tissue demarcating the entrance to the vagina, exhibits a high concentration of sensory free ends with a dense and shallow ramification and a high level of estrogen and androgenic receptors. In fact, therapies for dyspareunia that only specifically target the vulvar vestibule have been demonstrated to be effective [11,12]. Vestibulodynia (VBD), the most prevalent form of localized provoked vulvodynia, affects 10–16% of premenopausal women. It is marked by intense, burning pain confined to the vulvar vestibule triggered by light pressure (i.e., mechanical allodynia) and is associated with an increased perception of vulvar pain (i.e., mechanical hyperalgesia) [13]. The development and persistence of VBD have been linked to a multifactorial etiology. Factors such as recurrent candidiasis and vulvovaginal infections, hormonal changes, inflammation, allergies, genetic predisposition, and psychogenic vulnerability are believed to contribute to the sensitization of vestibular nerve fibers [14]. Vulvovaginal infections are frequently cited as inciting inflammatory events triggering the development of VBD, such as yeast infections, urinary tract infections, trichomonas, and vaginosis. Atrophic changes and vulnerability to vulvovaginal infections in the postmenopause stage could be a predisposing factor to the development of a VBD secondary to atrophy. Research employing both culture-based and molecular techniques indicates that postmenopausal women have a lower likelihood of vaginal colonization with Lactobacillus bacterial species compared to premenopausal women. [15]. This has been attributed to decreased serum estrogen, which reduces glycogen content in vaginal epithelial cells and limits the energy source for *Lactobacilli*, which are the main factor against vulvovaginal infections.

Tibolone (TIB) is a compound with estrogenic, progestogenic, and androgenic activity. TIB has a 3-keto-Δ5–10 steroid structure with 17α-ethynyl and 7α-methyl groups. It is very rapidly metabolized to 3α-hydroxy tibolone and 3β-hydroxy tibolone. These two metabolites are responsible for the estrogenic activity of tibolone, while a third metabolite, the Δ4-isomer of tibolone, has progestogenic and androgenic properties [16]. TIB treatment is recognized as a hormone replacement therapy (HRT) for postmenopausal women. It alleviates vasomotor symptoms and prevents bone loss without evidence of endometrial stimulation in postmenopausal women. However, it should not be used in patients with breast cancer or those who have survived breast cancer.

There is demonstration of TIB’s positive action on postmenopausal dyspareunia and vulvovaginal dryness [17,18,19]. These results might be explained by the activation of androgen and estrogen receptors; moreover, TIB has positive effects on the vaginal maturation index and induces the alleviation of the symptoms of atrophic vaginitis [20].

The aim of the present study was to assess the efficacy of the combination of TIB and an oral-specific *Lactobacilli* strain oral in combination with bovine lactoferrin (LF) as synergistic therapy for the treatment of VBD related to atrophy.

## 2. Materials and Methods

Women included in the study were postmenopausal with at least 1 year of amenorrhea, affected by vulvar burning/pain and introital dyspareunia. The cause of vulvodynia was mainly linked to postmenopausal estrogenic deficiency and frequent vaginitis/vaginosis associated, or not, with recurrent cystitis and/or other urinary symptoms. The first assessment symptoms were evaluated on a Visual Analog Scale (VAS): a numeric pain distress scale ranking from 0 (no pain) to 10 (unbearable pain) [21].

Women deemed eligible for the study underwent a pain evaluation test (cotton swab test) to assess sensitivity at six specific points around the vestibule, identifying pain locations on the vulva. The testing commenced at the thighs and proceeded medially to the vestibule. Resembling the positions on a clock face, the vestibule was tested at the 2:00, 4:00, 6:00, 8:00, 10:00, and 12:00 positions. At each position, the patient was asked to rate the pain as none (score 0), mild (score 1), moderate (score 2), or severe (score 3). Key exclusion criteria included the presence of clinically significant abnormal gynecological findings other than signs of vaginal atrophy; the use of concomitant hormonal medications, SERMs, or products expected to have estrogenic and/or antiestrogenic effects; or the use of hormone therapy. All participants received treatment with open-label, oral TIB 2.5 mg and *Lactobacilli* mixture (5 × 10^9^ CFU per capsule) including *Lactobacillus acidophilus* GLA-14 (BCCM/LMG Bacteria Collection, Gent, Belgium, LMG S-29159) and *Lactobacillus rhamnosus* HN001 in combination with bovine LF RCX™ (50 mg) (Respecta^®^). Each product was taken once daily for 90 days (3 months). During treatment, the women maintained a lifestyle comparable to their previous lifestyle. The present study protocol was reviewed and approved by the Institutional Review Board of V. Buzzi Hospital (approval No. 2023-02-034, date 18 March 2023), and written informed consent was obtained from all subjects involved in the study.

A statistical analysis was performed using nonparametric tests. The differences among groups of data, before (T0) and after (T1—90 d) the treatment, were tested by the Kruskal–Wallis test. The data are reported as mean ± standard deviation (SD). The differences observed have been considered statistically significant at *p* < 0.05.

## 3. Results

**Figure 1:**
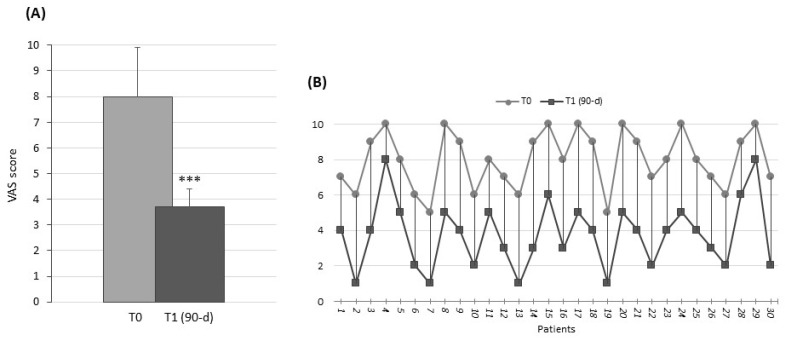
Visual Analog Scale (VAS). (**A**) Histograms show the mean values ± SD at T0 (light gray) and T1 (dark gray), after 90 d of treatment. *** *p* < 0.01. (**B**) Stacked line chart shows a comparison of pre- and post-treatment individual scores of the intervention patient group.

Out of the 35 enrolled women, 30 completed the 90 d treatment. The patients reported excellent tolerability to both TIB and the combination of probiotics and LF. The dropouts were related to personal reasons and did not involve issues related to the pharmacological collateral effects. No subject reported any negative side effects. After 90 d of therapy with TIB+ Respecta^®^, there was a statistically significant decrease from the baseline in the mean VAS for vulvar burning/pain, with the value changing from 8.0 ± 1.9 to 3.7 ± 0.7 (*p* < 0.01) (Figure 1).

**Figure 2:**
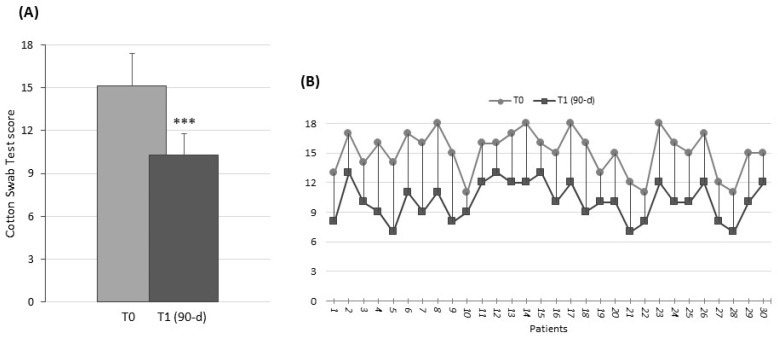
Cotton swab test—vaginal vestibular tissue sensitivity evaluation. (**A**) Histograms show the mean values ± SD at T0 (light gray) and T1 (dark gray), after 90 d of treatment. *** *p* < 0.01. (**B**) Stacked line chart shows comparison of pre- and post-treatment individual scores of the intervention patient group.

The patients also showed a statistically significant pain reduction in cotton swab test scores at the end of treatment (on a 0–3 range for the sum of all investigated area); the value was 10.3 ± 1.5 compared with 15.1 ± 2.3 at baseline (*p* < 0.01) (Figure 2).

Most patients reported feeling genital well-being and comfortably ‘wet’ after treatment as a result of symptom improvement. Almost all women reported a sense of well-being in the genital area, and many perceived a resurgence of vaginal secretions, somewhat like those experienced in the premenopause stage. External inspection by a gynecologist noted a reduction, and in some cases, absence of inflammation and hyperemia of the vaginal mucosa. Furthermore, among the enrolled patients, those who exhibited specific menopausal symptoms such as hot flashes, sweats, and nocturnal awakenings reported a significant reduction in the number of episodes.

## 4. Discussion

Vulvodynia is a pathological condition characterized by pain affecting the female external genitalia, manifesting with a range of symptoms including discomfort, pain upon genital contact, burning, itching, and stinging sensations. These symptoms can vary in duration, potentially persisting for months or even years, and often result in chronic pain particularly localized on the vulva and the tissues surrounding the vagina.

This study suggests that the combination of TIB+ Respecta^®^ may be effective in reducing symptoms related to vestibular pain and hypersensitivity in a postmenopausal setting.

Data suggest that lower genital tract complaints, more specifically defined as GSM, negatively affect a woman’s sexual health, relationships, and quality of life [22]. Despite its prevalence and widely available hormonal and non-hormonal therapies, only a minority of women are treated for GSM. Barriers to treatment include patient and provider discomfort discussing genitourinary and sexual complaints, lack of knowledge about therapies, and fear of serious side effects.

Regarding the role of estrogen in the development of this pathology, the presence of estrogen receptors on mast cells has been demonstrated. Mast cells, which are hyperactivated during vestibulitis, undergo continuous degranulation and release proinflammatory substances that perpetuate the pathological state. Consequently, therapy with topical estrogens is not indicated during the active phase of the disease, as it exacerbates inflammation. In addition, vaginal dryness and reduced lubrication, typical of the menopausal condition, contribute to the inflammatory aspect of the pathology.

The reduction in circulating estrogen levels observed in the postmenopause stage is responsible for a variety of symptoms ranging from hot flashes to night sweats and vaginal atrophy. There is scientific evidence that HRT in postmenopausal women reduces or eliminates most of these symptoms [10]. Despite the numerous benefits of HRT, the percentage of women using it is very low due to potential negative side effects, especially at the breast level. In our experience, many menopausal women who report vulvar burning or pain exhibit significant vestibular tenderness, with more noticeable atrophic changes in the vestibular area compared to the vagina. Additionally, patients whose symptoms are primarily vestibular, such as vulvar burning or pain, often do not experience relief when treated with systemic hormones or intravaginal preparations like vaginal tablets. This may be due to variations in the local effects of treatments based on their bioavailability and absorption site.

One strength of our study is the positive effect of TIB on vestibular pain and hypersensitivity. The vestibule has the capacity to develop overgrowth of nociceptive nerve fibers in a setting of estrogen and androgen deprivation. In fact, the vulvar vestibule expresses a high number of androgen receptors that are pivotal for maintaining trophic and functional actions, such as lubrication, nerve fiber density, and neurotransmission [23,24]. We can speculate that TIB improved vulva pain due to its properties that enable it to specifically stimulate the estrogen and androgen receptors. In addition, TIB may act indirectly to decrease sex-hormone-binding globulin concentrations and thereby increase the availability of testosterone. A healthy vaginal environment requires optimal vaginal microbiome conditions, and changes in the vaginal microorganisms during menopause are evident. The postmenopausal decrease in sexual hormones influences the vaginal microbiome, reducing the number of *Lactobacilli*. Some evidence highlighted a relationship between increased vaginal bacterial diversity, typical of dysbiosis, and poverty of *Lactobacilli* with complaints of vulvovaginal dryness and discomfort [25]. Vulvovaginal infections are frequently cited as an inciting inflammatory event triggering the development of VBD, and this is even more true for postmenopausal women. Another strength of our study is the synergistic action of Respecta^®^ to TIB. This can be related to the following three factors:

i. *Lactobacillus acidophilus* GLA-14 and *Lactobacillus rhamnosus* HN001 were found in the vagina after oral administration, and their positive effect on reducing the recurrence of vulvovaginal infections was demonstrated [26,27].
ii. LF can act as an antimicrobial and anti-inflammatory agent [28,29].
iii. Trophic action of TIB promotes the colonization of *Lactobacilli*.

## 5. Conclusions

In this preliminary study, both the analog scale and the cotton swab test screening confirm the relevant clinical data in resolving the aforementioned symptoms. Further studies on a larger number of women will be necessary to confirm these findings obtained with a therapeutic innovation combining TIB and a combination of *Lactobacilli*/LF, which may represent a good therapeutic strategy for advising postmenopausal women experiencing peripheral vulvodynia. The limitations of this study are the absence of follow-up, the small sample, and the lack of a control group. Nevertheless, no previous study has evaluated the vestibular effects of combination TIB plus probiotics for the treatment of postmenopausal vulvar pain. Our results are encouraging and should stimulate further research regarding this innovative pathophysiological neural mechanism of postmenopausal vulvar pain and vestibular hypersensitivity.

## Data Availability

All data underlying the study are available from the corresponding authors due to privacy. Despite this, data can be extrapolated from graphs.

## References

[1] MacNeill C. (2006). Dyspareunia. Obstet. Gynecol. Clin. N. Am..

[2] Oshinowo A., Ionescu A., Anim T.E., Lamvu G., Valovska A.T. (2016). Dyspareunia and Vulvodynia. Pelvic Pain Management.

[3] Sorensen J., Bautista K.E., Lamvu G., Feranec J. (2018). Evaluation and Treatment of Female Sexual Pain: A Clinical Review. Cureus.

[4] Kim H.-K., Kang S.-Y., Chung Y.-J., Kim J.-H., Kim M.-R. (2015). The Recent Review of the Genitourinary Syndrome of Menopause. J. Menopausal Med..

[5] The NAMS 2020 GSM Position Statement Editorial Panel (2020). The 2020 Genitourinary Syndrome of Menopause Position Statement of The North American Menopause Society. Menopause.

[6] Wasnik V.B., Acharya N., Mohammad S. (2023). Genitourinary Syndrome of Menopause: A Narrative Review Focusing on Its Effects on the Sexual Health and Quality of Life of Women. Cureus.

[7] Portman D.J., Gass M.L.S. (2014). Consensus Conference Panel Genitourinary Syndrome of Menopause: New Terminology for Vulvovaginal Atrophy from the International Society for the Study of Women’s Sexual Health and the North American Menopause Society. Menopause.

[8] Palacios S., Mejía A., Neyro J.L. (2015). Treatment of the Genitourinary Syndrome of Menopause. Climacteric.

[9] Angelou K., Grigoriadis T., Diakosavvas M., Zacharakis D., Athanasiou S. (2020). The Genitourinary Syndrome of Menopause: An Overview of the Recent Data. Cureus.

[10] Lara L.A., Cartagena-Ramos D., Figueiredo J.B., Rosa-E-Silva A.C.J., Ferriani R.A., Martins W.P., Fuentealba-Torres M. (2023). Hormone Therapy for Sexual Function in Perimenopausal and Postmenopausal Women. Cochrane Database Syst. Rev..

[11] Goetsch M.F., Lim J.Y., Caughey A.B. (2015). A Practical Solution for Dyspareunia in Breast Cancer Survivors: A Randomized Controlled Trial. J. Clin. Oncol..

[12] Murina F., Graziottin A., Felice R., Di Francesco S. (2016). Coital Pain in the Elderly: Could a Low Dose Estriol Gel Thrill the Vulvar Vestibule?. Eur. J. Obstet. Gynecol. Reprod. Biol..

[13] Graziottin A., Murina F., Graziottin A., Murina F. (2011). What Women with Vulvodynia Complain of: Evaluation of Vulvar Pain. Clinical Management of Vulvodynia: Tips and Tricks.

[14] (2023). Vulvodynia. J. Midwifery Womens Health.

[15] Brotman R.M., Shardell M.D., Gajer P., Fadrosh D., Chang K., Silver M.I., Viscidi R.P., Burke A.E., Ravel J., Gravitt P.E. (2014). Association between the Vaginal Microbiota, Menopause Status, and Signs of Vulvovaginal Atrophy. Menopause.

[16] Formoso G., Perrone E., Maltoni S., Balduzzi S., Wilkinson J., Basevi V., Marata A.M., Magrini N., D’Amico R., Bassi C. (2016). Short-Term and Long-Term Effects of Tibolone in Postmenopausal Women. Cochrane Database Syst. Rev..

[17] Huang K.-E., Baber R., Asia Pacific Tibolone Consensus Group (2010). Updated Clinical Recommendations for the Use of Tibolone in Asian Women. Climacteric.

[18] Casiano Evans E.A., Hobson D.T.G., Aschkenazi S.O., Alas A.N., Balgobin S., Balk E.M., Dieter A.A., Kanter G., Orejuela F.J., Sanses T.V.D. (2023). Nonestrogen Therapies for Treatment of Genitourinary Syndrome of Menopause: A Systematic Review. Obstet. Gynecol..

[19] Fait T. (2019). Tibolon—The Only One Member of STEARs Group. Cas. Lek. Cesk.

[20] Malik R., Meghana Reddy P. (2023). Effectiveness of Tibolone in Relieving Postmenopausal Symptoms for a Short-Term Period in Indian Women. J. Obstet. Gynaecol. India.

[21] Schlaeger J.M., Glayzer J.E., Villegas-Downs M., Li H., Glayzer E.J., He Y., Takayama M., Yajima H., Takakura N., Kobak W.H. (2023). Evaluation and Treatment of Vulvodynia: State of the Science. J. Midwifery Womens Health.

[22] Nappi R.E., Martini E., Cucinella L., Martella S., Tiranini L., Inzoli A., Brambilla E., Bosoni D., Cassani C., Gardella B. (2019). Addressing Vulvovaginal Atrophy (VVA)/Genitourinary Syndrome of Menopause (GSM) for Healthy Aging in Women. Front. Endocrinol..

[23] Traish A.M., Vignozzi L., Simon J.A., Goldstein I., Kim N.N. (2018). Role of Androgens in Female Genitourinary Tissue Structure and Function: Implications in the Genitourinary Syndrome of Menopause. Sex. Med. Rev..

[24] Maseroli E., Vignozzi L. (2020). Testosterone and Vaginal Function. Sex. Med. Rev..

[25] Hummelen R., Macklaim J.M., Bisanz J.E., Hammond J.-A., McMillan A., Vongsa R., Koenig D., Gloor G.B., Reid G. (2011). Vaginal Microbiome and Epithelial Gene Array in Post-Menopausal Women with Moderate to Severe Dryness. PLoS ONE.

[26] De Alberti D., Russo R., Terruzzi F., Nobile V., Ouwehand A.C. (2015). Lactobacilli Vaginal Colonisation after Oral Consumption of Respecta^®^ Complex: A Randomised Controlled Pilot Study. Arch. Gynecol. Obstet..

[27] Russo R., Superti F., Karadja E., De Seta F. (2019). Randomised Clinical Trial in Women with Recurrent Vulvovaginal Candidiasis: Efficacy of Probiotics and Lactoferrin as Maintenance Treatment. Mycoses.

[28] Levay P.F., Viljoen M. (1995). Lactoferrin: A General Review. Haematologica.

[29] Artym J., Zimecki M. (2021). Antimicrobial and Prebiotic Activity of Lactoferrin in the Female Reproductive Tract: A Comprehensive Review. Biomedicines.

